# Synthesis, characterization and initial evaluation of 5-nitro-1-(trifluoromethyl)-3*H*-1λ^3^,2-benziodaoxol-3-one

**DOI:** 10.3762/bjoc.10.1

**Published:** 2014-01-02

**Authors:** Nico Santschi, Roman C Sarott, Elisabeth Otth, Reinhard Kissner, Antonio Togni

**Affiliations:** 1 Department of Chemistry and Applied Biosciences, Swiss Federal Institute of Technology (ETH Zürich), Wolfgang-Pauli-Str. 10, CH-8093 Zürich, Switzerland

**Keywords:** cyclic voltammetry, differential scanning calorimetry, electrophilic trifluoromethylation, ^19^F NMR kinetics, nitration, organo-fluorine

## Abstract

The synthesis of 5-nitro-1-(trifluoromethyl)-3*H*-1λ^3^,2-benziodaoxol-3-one (**3**), a hypervalent-iodine-based electrophilic trifluoromethylating reagent, is described. Whereas considerations based on cyclic voltammetry and X-ray structural properties would predict an inferior reactivity when compared to the non-nitrated derivative **2**, ^19^F NMR kinetic studies showed that this new derivative is almost one order of magnitude more reactive. Furthermore, differential scanning calorimetry measurements indicated that, in addition, it is also safer to handle.

## Introduction

**Figure 1:**
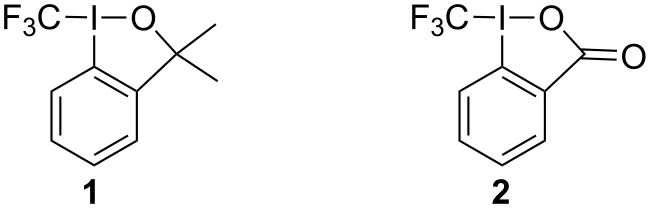
Electrophilic trifluoromethylating agents **1** and **2**.

Since the advent of the hypervalent-iodine-based electrophilic trifluoromethylation reagents **1** and **2** in 2006 they have found widespread application in organic synthesis (Figure 1) [1–4]. Recently, increasing interest has been directed towards the photocatalytic one-electron reduction of these compounds in order to generate the highly electrophilic trifluoromethyl radical [5–7].

For example, Gouverneur et al. have highlighted the regioselective allylic trifluoromethylation employing reagent **2**, [Ru(bpy)_3_]Cl_2_ and allylsilanes as substrates [7]. In this context, also extensive electrochemical characterizations of various electrophilic trifluoromethylating agents are now available [8]. These studies reported cathodic peak potentials versus the Ag/Ag^+^ pair of −1.82 V and −1.10 V for **1** and **2**, respectively. Therefore, we contemplated the possibility of enhancing the reactivity of reagent **2** by rendering its scaffold less electron-rich. This was to be achieved through the introduction of a strongly electron-withdrawing substituent in *para*-position to the iodine atom, potentially also leading to a more positive standard reduction potential. Therefore, based on a recent report by Togo et al. [9], the nitration of the reagent scaffold to afford the new derivative 5-nitro-1-(trifluoromethyl)-3*H*-1λ^3^,2-benziodaoxol-3-one (**3**) was taken into consideration.

## Results and Discussion

**Scheme 1:**
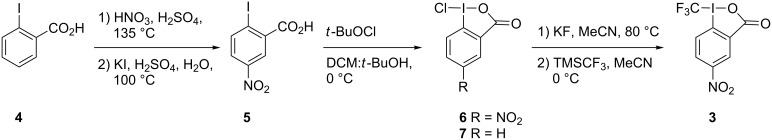
Synthesis of 5-nitro-1-(trifluoromethyl)-3*H*-1λ^3^,2-benziodaoxol-3-one (**3**).

The direct nitration of 2-iodobenzoic acid (**4**) has been reported before and was carried out on a 10 gram scale in a mixture of HNO_3_ and H_2_SO_4_ at 135 °C (Scheme 1) [10]. Reductive work-up using an aqueous acidic solution of potassium iodide furnished the desired product **5** in 11–46% yield (29% average yield of four experiments). Subsequent oxidation was centered on an improved synthesis of reagent **2** employing trichloroisocyanuric acid (TCICA) as cheap, stoichiometric oxidant, as recently reported from our laboratory [11]. Although compound **5** was readily converted to **6** in hot MeCN with TCICA in 90% yield an alternative protocol was found advantageous in terms of overall reaction time, solvent consumption and time required to dry the product. Consequently, treatment of **5** in a mixture of DCM/*t*-BuOH 9:1 at 0 °C with *t*-BuOCl under exclusion of light led to the immediate precipitation of 1-chloro-5-nitro-3*H*-1λ^3^,2,benziodaoxol-3-one (**6**) in 81–93% yield (89% average yield of five experiments). This step was routinely carried out on a 3 gram scale in less than 5 minutes. Finally, introduction of the trifluoromethyl group was achieved by stirring **6** and anhydrous KF in hot MeCN, followed by treatment with Ruppert–Prakash reagent (TMSCF_3_) at 0 °C yielding 10–35% of the desired product **3** as a pale yellow solid. Unfortunately, a slightly more economic route employing KOAc instead of KF was not feasible. Additionally, the purity of the product obtained was dependent on the grade of KF that was used. Therefore, spray-dried KF (99%) was further flame-dried under high vacuum immediately before use.

**Figure 2:**
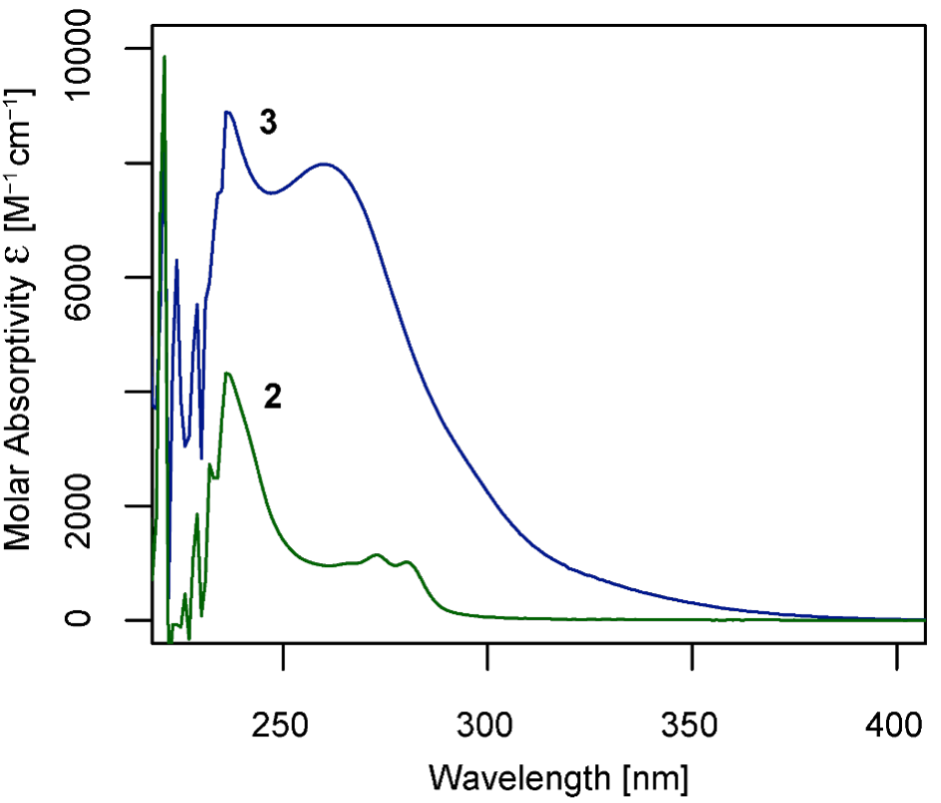
UV–vis spectra of reagents **2** (green) and **3** (blue) in DMSO/H_2_O 1:1.

Inspection of the ^13^C NMR chemical shift of the quaternary phenyl carbon attached to iodine (C_Ar-I_) in **5** and comparison to literature data available for 2-iodobenzoic acid (**4**) indeed confirmed the installation of an electron-poor center [12]. Hence, in the parent acid **4** C_Ar-I_ gives rise to a signal at 94.1 ppm and in **5** the corresponding carbon atom is deshielded by 9.6 ppm and observed at 103.7 ppm. The same observation holds true for **6** and its unmodified analogue **7** (104.0 ppm versus 94.1 ppm, Δδ = 9.9 ppm) as well as for **3** and **2** (121.8 ppm versus 114.8, Δδ = 7 ppm). Furthermore, this comparison highlights that the replacement of the chlorine atom by the CF_3_ group to a certain degree compensates the electronic effects imposed by the NO_2_ group as indicated by the lessened extent of deshielding. Nitration also had a distinct effect on the electronic spectrum of the final reagent. Solutions of **2** and **3** in DMSO/H_2_O 1:1 were studied at concentrations of 9.7 × 10^−5^ M and 9.1 × 10^−5^ M, respectively. A graphic representation of the molar absorptivity ε vs. the corresponding wavelengths is presented in Figure 2. Parent reagent **2** features an absorbance maximum at λ_max_ = 237 nm (ε = 4.3 × 10^3^ M^−1^ cm^−1^) and two resolved sidebands at 272 nm (ε = 1.1 × 10^3^ M^−1^ cm^−1^) and 280 nm (ε = 1.0 × 10^3^ M^−1^ cm^−1^), respectively. In contrast, the nitrated product **3** exhibits a maximum λ_max_ = 236 nm (ε = 8.9 × 10^3^ M^−1^ cm^−1^) and features a broad sideband ranging from 260 nm (ε = 8.0 × 10^3^ M^−1^ cm^−1^) up to 350 nm, hence accounting for its pale yellow coloration. Generally, these observations are in line with data reported on the analogous pair of iodobenzene and 1-iodo-4-nitrobenzene [13–14]. However, whereas the broad sideband in 1-iodo-4-nitrobenzene is usually observed around 300 nm, a strong hypsochromic shift of roughly 40 nm is observed for **3**. This can be explained by the installation of the hypervalent bond and the presence of the strongly electron-withdrawing CF_3_ group, as also indicated by the carbon chemical shift analysis.

**Figure 3:**
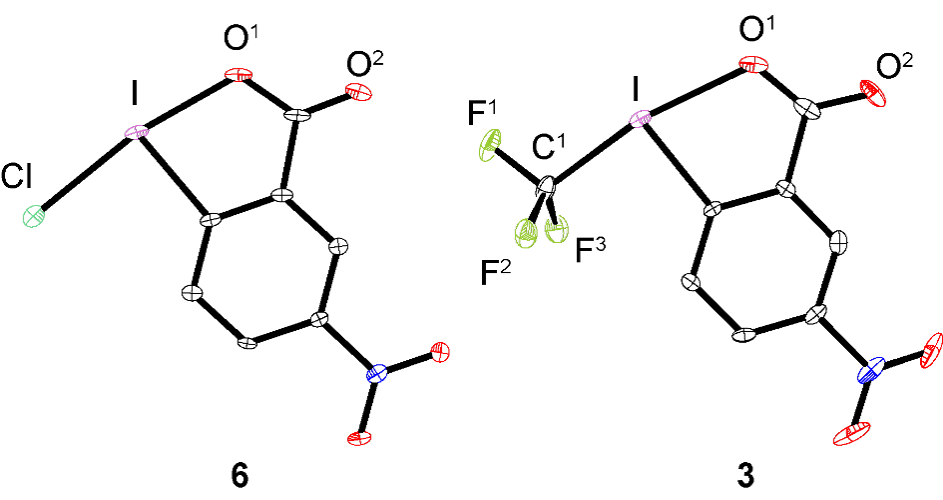
ORTEP representation of X-ray structures **6** and **3**. Thermal ellipsoids set to 30% probability. Hydrogen atoms omitted for clarity.

Compounds **6** and **3** were also characterized by means of X-ray crystallography. Due to the very poor solubility of **6** in most common solvents, suitable single crystals for solid-state analysis were grown from a saturated solution in hot MeCN that was slowly cooled to −20 °C. Similarly, suitable crystals of reagent **3** were obtained from a boiling solution in bromobenzene. Whereas **6** crystallizes in plain sheets, **3** forms zig-zag-shaped layers. ORTEP representations of compounds **6** and **3** are given in Figure 3. Selected bond lengths and angles of **6**, **3** and the parent compounds **7**, **2** are summarized in Table 1.

**Table 1:** Selected bond lengths and angles for compounds **2**, **3**, **6**, **7**.

Entry	I–X [Å] X = Cl, CF_3_	I–O^1^ [Å]	O^1^–I–X [°] X = Cl, CF_3_

**7**^a^	2.461(1)	2.091(3)	171.96(8)
**6**	2.4409(11)	2.148(3)	170.52(8)
**2**^b^	2.219(4)	2.283(2)	170.49(12)
**3**	2.200(6)	2.306(4)	169.01(17)

^a^Reference [15]. ^b^Reference [1].

Both structures are stabilized by intermolecular hydrogen bonds (NO_2_···H–Ar) [16] and by short intermolecular I–O^2^ contacts, as typical for this class of compounds (see Supporting Information File 1). These structural findings also account for the slow dissolution kinetics observed.

Generally, the introduction of the NO_2_ functionality is associated with a shortening of the I–X bond (Δ = 0.0201 Å for X = Cl; Δ = 0.019 Å for X = CF_3_) and concomitant elongation of the I–O^1^ bond (Δ = 0.057 Å for X = Cl; Δ = 0.023 Å for X = CF_3_). Furthermore, the O^1^–I–X angles are comparable for all four structures being 1 to 1.5° smaller for the nitro-substituted compounds. When comparing the same unit I–X within the analogous compounds **2** and **3**, a shorter bond would intuitively correspond to a stronger bond. Therefore, based on simple geometrical parameters, reagent **3** was expected to be slightly less reactive compared to **2**, meaning that reductive cleavage of the I–CF_3_ bond should display a higher activation barrier.

**Figure 4:**
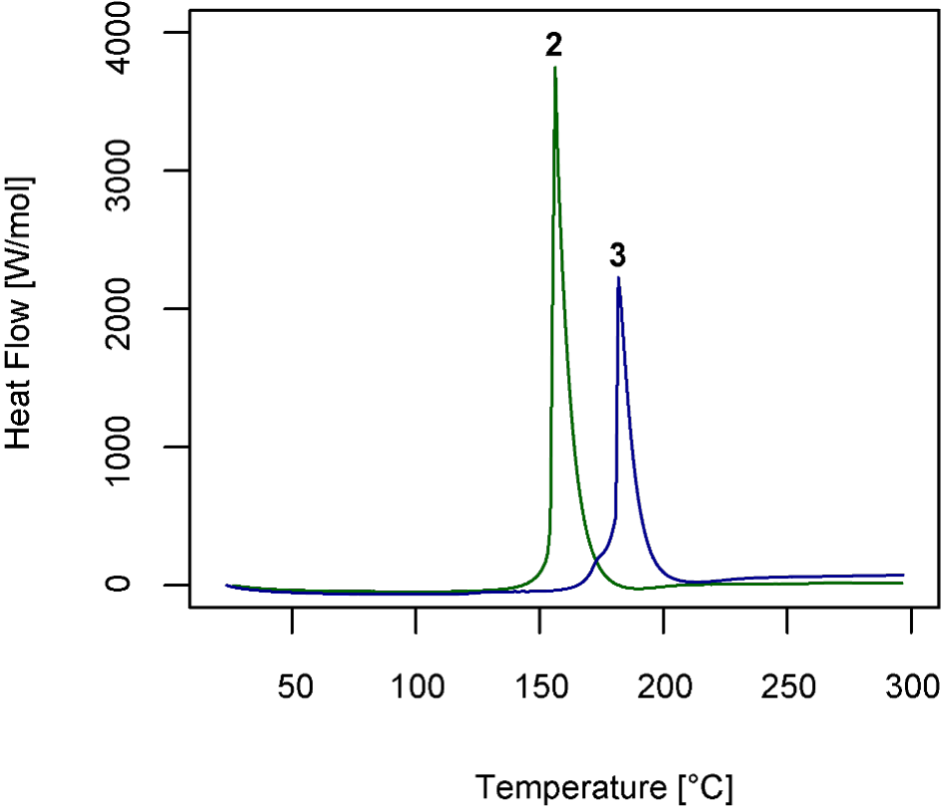
DSC traces obtained for **2** (green) and **3** (blue).

Recently, differential scanning calorimetry (DSC) measurements for reagent **2** and its precursors were published indicating a decomposition energy of 159 kJ/mol (502 J/g) for **2** [17]. Furthermore, a Koenen test suggested explosiveness. Consequently in light of these results, DSC measurements of **2** and **3** were carried out in order to assess the thermal stability of the new derivative. These measurements were performed in punctured Al_2_O_3_ pans with a heating rate of 10 K/min. For reagent **2**, a decomposition energy of 138 kJ/mol (438 J/g, slightly lower than reported) with a maximum heat flow of 3.7 kW/mol (11.9 W/g) was found. Intriguingly, compound **3** features a significantly lower decomposition energy of only 99 kJ/mol (273 J/g) and a maximum heat flow of 2.2 kW/mol (6.2 W/g), therefore indicating that it should be safer to handle than parent compound **2** (Figure 4). The higher temperature of decomposition of 25 °C for **3**, as measured by the difference of the corresponding temperatures at peak heat flow, is believed to directly result from the additional stabilization of **3** by intermolecular H-bonding (vide supra).

**Figure 5:**
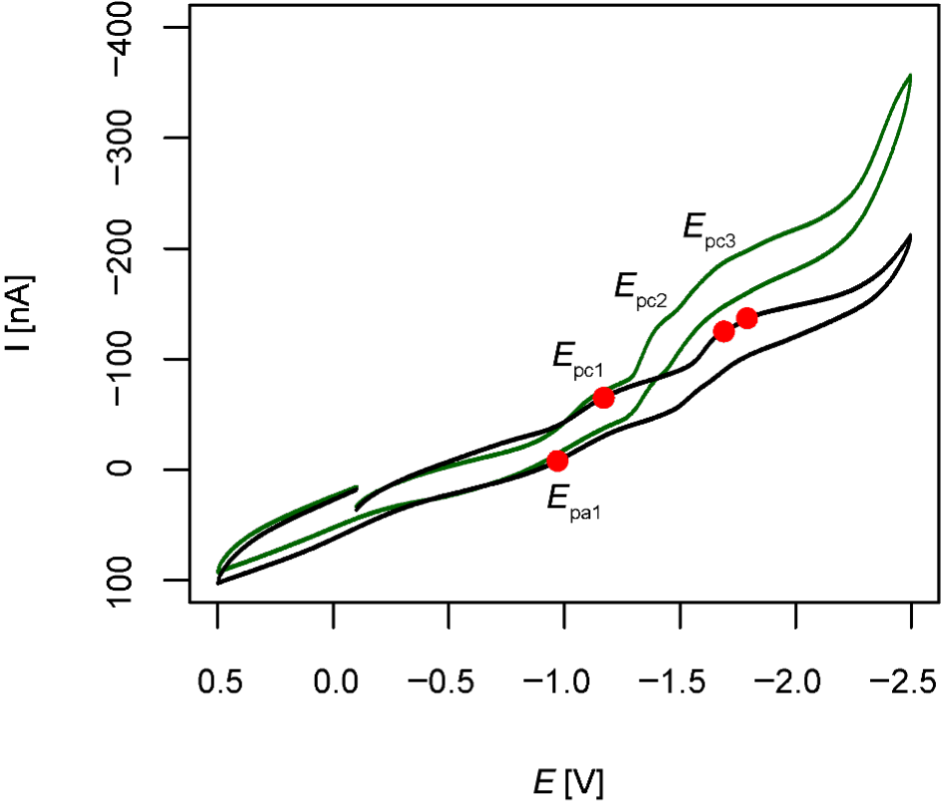
Cyclic voltammetry of **3** (1 mM, black) and of a mixture of **3** (1 mM) and methyl 2-iodo-5-nitrobenzoate (1 mM, green) in anhydrous MeCN + 0.1 M Bu_4_NBF_4_, platinum electrode, scan rate = 0.1 V/s.

Additionally, in order to test the initial hypothesis that nitration may lead to a more positive reduction potential, cyclic voltammetry (CV) measurements were carried out. These experiments were conducted under an atmosphere of nitrogen in anhydrous MeCN with Bu_4_NBF_4_ (0.1 M) as supporting electrolyte. A platinum working electrode and a Ag/Ag^+^ (0.1 M AgBF_4_ in MeCN) reference electrode were employed with a scan rate of 0.1 V/s. Analysis of the resulting cyclic voltammogram of a 1 mM solution of **3** revealed three quasi-reversible processes with cathodic peak potentials (*E*_pc_) of −1.2, −1.7 and −1.8 V, respectively (Figure 5). Subsequently, through addition of methyl 2-iodo-5-nitrobenzoate (1 mM), the first wave could be unambiguously identified as the reduction of the hypervalent bond. Therefore, the more negative waves most likely correspond to the reduction of the nitro group and/or further reduction of iodine as reported in CV studies carried out on bis(acetoxy)iodobenzene [18].

For the first wave *E*_pc1_ = −1.17 and *E*_pa1_ = −0.97 were determined, indicating a standard reduction potential *E*^0^_1/2_ = −1.1 V vs the Ag/Ag^+^ pair. These values are comparable to the ones reported for reagent **2** (*E*_pc_ = −1.1 V vs Ag/Ag^+^, scan rate 0.2 V/s, MeCN) [8]. Therefore, nitration did not influence the standard reduction potential significantly and **3** should behave in a comparable manner as **2**. These electrochemical findings are in line with the expectations of reactivity based on the X-ray structural considerations.

**Figure 6:**
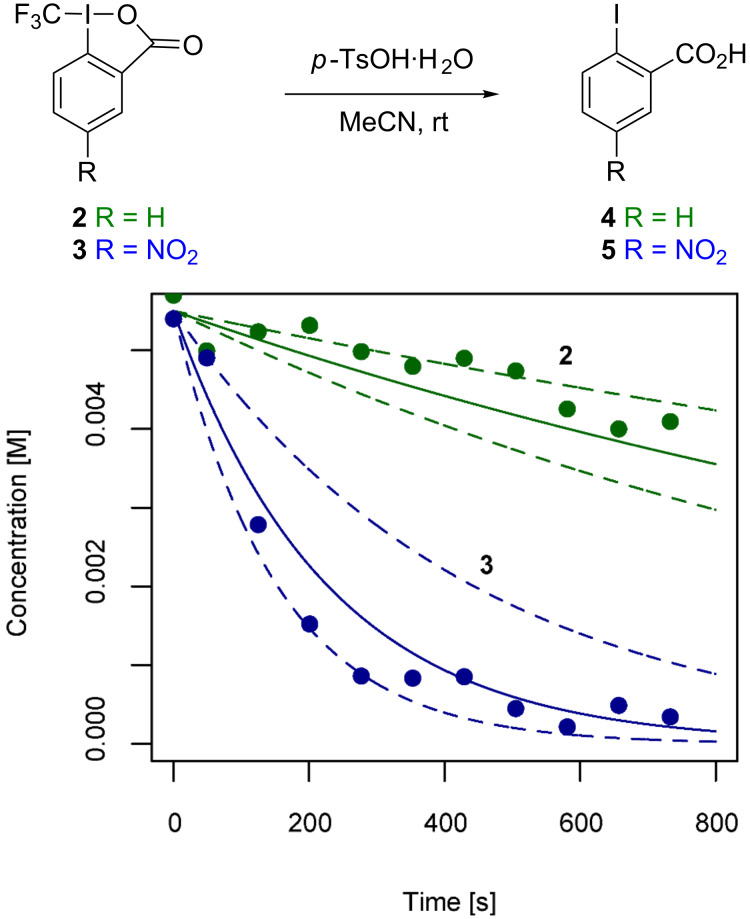
Sample kinetic traces for the decomposition of **2** (green) and **3** (blue) to **4** and **5**, respectively, upon reaction with *p*-TsOH in MeCN. Solid lines represent the average decay (*k*_decomp_) based on 6 individual measurements and dashed lines are decays based on *k*_decomp_ ± σ.

Finally, the decomposition kinetics of both reagents in a model system were studied providing insights into their relative reactivities. To this end, the reaction of **2** and **3** with *p*-toluenesulfonic acid monohydrate to **4** and **5**, respectively, was monitored in MeCN by means of ^19^F NMR spectroscopy (Figure 6). Limitations arose from the poor solubility of **3** (1 mg in 0.25 mL MeCN) and, in the case of **2**, from line broadening resulting after the addition of the substrate, hence necessitating recording of 6 kinetic traces per reagent. Reagent **3** exhibits an exponential decay whereas the concentration of **2**, on the same time scale, decreases linearly. Therefore, rate data for **3** were obtained by curve fitting according to an exponential rate law and the method of initial rates was applied to reagent **2**. Subsequently, the initial rates were transformed to the corresponding first-order rate constants under the assumption that for both reagents the same mechanism of decay is operational. Indeed, the data imply that **3** (*k*_decomp_ = 4.43 ± 2.15 × 10^−3^ s^−1^) is less stable under the reaction conditions chosen and decays almost one order of magnitude faster than reagent **2** (*k*_decomp_ = 5.46 ± 2.23 × 10^−4^ s^−1^).

## Conclusion

In conclusion, the new derivative **3** having an additional nitro functionality as compared to reagent **2** was successfully prepared and characterized. The resulting electronic modification was studied by analyzing carbon chemical shifts, UV–vis spectra and cyclic voltammetry. Whereas the first two methods clearly indicated the installation of an electron-poor center adjacent to iodine, cyclic voltammetry delivered a standard reduction potential comparable to the one of **2**. Notably, differential scanning calorimetric measurements indicate that the new reagent is thermally more stable. In presence of a proton source, however, increased lability of **3** was noted such that it decayed almost one order of magnitude faster than **2** under similar conditions. Therefore, the incorporation of an electron-withdrawing group into the reagent scaffold leads to an enhanced thermal stability that is paralleled by increased lability of the I–CF_3_ bond under acidic conditions. However, the major drawback of the new modification is its low solubility, a problem that is currently being addressed in our group.

## Experimental

**2-Iodo-5-nitrobenzoic acid (5):** During this preparation up to 25 mmol I_2_ are formed. Therefore, the synthesis has to be conducted in a well-ventilated fume cupboard. Prepared according to literature procedure [10]. 2-Iodobenzoic acid (**4**, 10 g, 40.3 mmol, 1 equiv) was added to a mixture of HNO_3_ (35 mL, 65%) and H_2_SO_4_ (85 mL, 95%). The solution was heated to 135 °C and stirred for 1 h. Subsequently, the resulting brown slurry was poured onto ice and the grey precipitate was filtered off and washed with copious amounts of water. Then, the filtrate was suspended in water (100 mL), heated to 100 °C and treated with a solution of KI (8.5 g, 51.2 mmol, 1.3 equiv) and H_2_SO_4_ (5 drops) in water (10 mL) over the course of 1 h. Finally, the brown suspension was filtered hot and washed with water to afford the pure product (5 g, 42%) as a brown solid. If impure product is obtained, boiling with water followed by hot filtration can be applied. Mp 195**–**198 °C; ^1^H NMR (300 MHz, DMSO-*d*_6_) δ 8.40 (d, *J* = 2.7 Hz, 1H), 8.29 (d, *J* = 8.6 Hz, 1H), 8.01 (dd, *J* = 8.6, 2.7 Hz, 1H), 4.00 (s, 1H); ^13^C NMR (75 MHz, DMSO-*d*_6_) δ 166.5 (*C*O_2_), 147.3 (*C*NO_2_), 142.4 (*C*H *ortho* to CI), 138.2 (*C*CO_2_), 126.1 (*C*H *para* to CCO_2_), 124.1 (*C*H *ortho* to CCO_2_), 103.7 (*C*I).

**1-Chloro-5-nitro-3*H*-1λ^3^,2-benziodaoxol-3-one (6):** To a solution of 2-iodo-5-nitrobenzoic acid (**5**, 1 g, 3.4 mmol, 1 equiv) in DCM/*t*-BuOH 9:1 (12 mL) at 0 °C *t*-BuOCl (0.42 mL, 3.7 mmol, 1.1 equiv) was added in one portion, taking care to shield this reagent from light. Stirring was continued for 20 min, the suspension was filtered and the filtrate washed with DCM to afford pure product (0.95 g, 86%) as a yellow solid. X-ray quality crystals were obtained by recrystallization from boiling MeCN. Mp 225 °C (dec); ^1^H NMR (400 MHz, DMSO-*d*_6_) δ 8.38 (d, *J* = 2.1 Hz, 1H), 8.26 (d, *J* = 8.6 Hz, 1H), 7.99 (dd, *J* = 8.5, 2.2 Hz, 1H); ^13^C NMR (101 MHz, DMSO-*d*_6_) δ 166.3 (*C*O_2_), 147.3 (*C*NO_2_), 142.5 (*C*H *ortho* to CI), 137.9 (*C*CO_2_), 126.2 (*C*H *para* to CCO_2_), 124.2 (*C*H *ortho* to CCO_2_), 104.1 (*C*I); HRMS–EI (*m*/*z*): [M]^+^ calcd for C_7_H_3_ClINO_4_, 326.8795; found, 326.8775; Anal. calcd for C_7_H_3_ClINO_4_: C, 25.68; H, 0.92; N, 4.28; found: C, 25.42; H, 1.02; N, 4.28.

**5-Nitro-1-(trifluoromethyl)-3*H*-1λ^3^,2-benziodaoxol-3-one (3):** Spray-dried KF (0.61 g, 10.3 mmol, 3 equiv) was flame-dried under high vacuum, then suspended with 1-chloro-5-nitro-3*H*-1λ^3^,2-benziodaoxol-3-one (**6**, 1 g, 3.1 mmol, 1 equiv) in dry MeCN (125 mL) under argon and stirred at 75 °C for 3.5 h, leading to the precipitation of a white solid. The suspension was cooled to 0 °C, TMSCF_3_ (0.55 mL, 3.7 mmol, 1.2 equiv) was added and stirring continued for 2 h. The reaction mixture was filtered through a Celite pad. Subsequently, the organic layer was washed with brine (50% sat), dried over Na_2_SO_4_, and concentrated under reduced pressure to turbidity. Filtration and washing with MeCN afforded the pure product (0.35 g, 32%) as a pale yellow solid. If impure product is obtained, recrystallization form MeCN may be applied. X-ray quality crystals were obtained by recrystallization from boiling bromobenzene. Mp 163 °C (dec); ^1^H NMR (300 MHz, DMSO-*d*_6_) δ 8.68 (d, *J* = 2.6 Hz, 1H), 8.64 (dd, *J* = 8.9, 2.7 Hz, 1H), 8.08 (d, *J* = 8.8 Hz, 1H); ^13^C NMR (75 MHz, DMSO-*d*_6_) δ 164.2 (*C*O_2_), 149.9 (*C*NO_2_), 134.8 (*C*CO_2_), 130.0 (q, *J*_CF_ = 3.7 Hz, *C*H *ortho* to CI), 129.3 (*C*H *para* to CCO_2_), 125.7 (*C*H *ortho* to CCO_2_), 121.8 (*C*I), 106.6 (q, *J*_CF_ = 383.0 Hz, *C*F_3_); ^19^F NMR (282 MHz, DMSO-*d*_6_) δ −32.6; HRMS–EI (*m*/*z*): [M + H]^+^ calcd for C_8_H_3_F_3_INNaO_4_, 383.8951; found, 383.8961; Anal calcd for C_8_H_3_F_3_INO_4_: C, 26.62; H, 0.84; N, 3.88; found: C, 26.67; H, 0.93; N, 4.08.

## Supporting Information

File 1 Experimental details, crystallographic data (selected intermolecular bond lengths) as well as a description of the ^19^F NMR monitoring experiments and data analysis.
