# Supplementary material for: Synthesis, characterization and initial evaluation of 5-nitro-1-(trifluoromethyl)-3H-1λ3,2-benziodaoxol-3-one
Source: Beilstein J Org Chem. 2014 Jan 2;10:1–6. doi: 10.3762/bjoc.10.1 (PMC3896278; doi:10.3762/bjoc.10.1)
Supplement: File 1 — Experimental details, crystallographic data (selected intermolecular bond lengths) as well as a description of the 19F NMR monitoring experiments and data analysis. [file Beilstein_J_Org_Chem-10-01-s001.pdf]

Supporting Information

for

Synthesis, characterization and initial evaluation of 5-nitro-1-(trifluoromethyl)-3*H*-1λ^3,2-benziodaoxol-3-one

Nico Santschi, Roman C. Sarott, Elisabeth Otth, Reinhard Kissner and Antonio Togni*

Address: Department of Chemistry and Applied Biosciences, Swiss Federal Institute of Technology (ETH Zürich), Wolfgang-Pauli-Str. 10, CH-8093 Zürich, Switzerland

Email: Antonio Togni* - atogni@ethz.ch

* Corresponding author

Experimental details, crystallographic data (selected intermolecular bond lengths) as well as a description of the ^{19}F NMR monitoring experiments and data analysis

Table of Contents

Experimental details..S2
Crystallographic details S3
^{19}F NMR monitoring setup S7
Cyclic voltammetry spectrum of **3**...S8
References..S9

Experimental details

NMR spectra were recorded on Bruker Avance III HD Nanobay-300 and Avance III HD Nanobay-400 spectrometers. ^1H and ^{13}C chemical shifts are referenced to residual solvent signals. ^{19}F chemical shifts are referenced to external $CFCl_3$. Mass spectra (EI) were measured by the MS-service of the Laboratory of Organic Chemistry (ETH Zurich). Elemental analyses were carried out by the Laboratory of Microelemental Analysis (ETH Zurich). Melting points were determined on a Büchi Melting Point B-540 apparatus and are uncorrected. UV–vis spectra were obtained with an Analytik Jena Specord 200 spectrometer. DSC traces were recorded on a NETZSCH DSC 404 apparatus. **CV** was performed on a Metrohm Autolab PGSTAT128N with a Pt 100 μm BAS working electrode and a Ag/Ag^+ (0.1 M $AgBF_4$ in MeCN) reference electrode. X-ray intensity data of single crystals glued to a glass capillary were collected at the given temperature (usually 100 K) on a Bruker SMART APEX platform with CCD detector and graphite monochromated MoK_α radiation (λ = 0.71073 Å). The program SMART served for data collection; integration was performed with the software SAINT (SAINT+, Software for CCD Diffractometers, v. 6.01; Bruker AXS, Inc., Madison, WI, 2001 and SAINT, v. 6.02). The structures were solved by direct methods or Patterson methods, respectively, using the program SHELXS-97 [1]. The refinement and all further calculations were carried out using SHELXL-97 [2]. All non-hydrogen atoms were refined anisotropically using weighted full-matrix least-squares on F^2. The hydrogen atoms were included in calculated positions and treated as riding atoms using SHELXL default parameters. In the end absorption correction was applied (SADABS) [3] and weights were optimized in the final refinement cycles. The standard uncertainties (s.u.) are rounded according to the "Notes for Authors" of *Acta Crystallographica* [4].

Crystallographic details

The crystal structures of **6** and **3** have been deposited at the Cambridge Crystallographic Data Center as CCDC 958118 and CCDC 958117, respectively.

Crystallographic data for compound 6

CCDC	958118	
Empirical formula	$C_7H_3ClINO_4$	
Formula Weight	327.45	
Temperature	100(2) K	
Wavelength	0.71073 Å	
Crystal system, space group	Orthorhombic,	*Pnma*
Unit cell dimensions	a = 9.8332(10) Å	α = 90°
	b = 6.3159(6) Å	β = 90°
	c = 14.5737(14) Å	γ = 90°
Volume	905.11(15) $Å^3$	
Z, Calculated density	4, 2.403 Mg/m^3	
Absorption coefficient	3.818 mm^{-1}	
F(000)	616	
Crystal size	0.17 x 0.14 x 0.08 mm	
Data collection	Siemens SMART PLATFORM with CCD Detector<br>Graphite monochromator	
Detector distance	50 mm	
Method; exposure time/frame	omega-scans; t = 10 sec	
Solution by	direct methods	
Refinement method	full matrix least-squares on F^2, SHELXTL	
Theta range for data collection	2.50 to 29.13°	
Limiting indices	-13<=h<=13, -8<=k<=8, -19<=l<=19	
Reflections collected / unique	12511 / 1323 [R(int) = 0.0312]	
Completeness to θ = 29.13	100.0%	
Absorption correction	Empirical	
Max. and min. transmission	0.7601 and 0.5681	
Refinement method	Full-matrix least-squares on F^2	
Data / restraints / parameters	1323 / 0 / 85	
Goodness-of-fit on F^2	0.748	
Final R indices [I>2σ(I)]	R_1 = 0.0213, wR_2 = 0.0793	
R indices (all data)	R_1 = 0.0247, wR_2 = 0.0855	
Largest diff. peak and hole	1.822 and -1.574 e.$Å^{-3}$	

Crystallographic data for compound 3

CCDC	958117	
Empirical formula	$C_8H_3F_3INO_4$	
Formula Weight	361.01	
Temperature	100(2) K	
Wavelength	0.71073 Å	
Crystal system, space group	Orthorhombic,	*Pbca*
Unit cell dimensions	a = 11.0917(5) Å	α = 90°
	b = 7.2595(4) Å	β = 90°
	c = 24.7955(12) Å	γ = 90°
Volume	1996.54(17) $Å^3$	
Z, Calculated density	8, 2.402 Mg/m^3	
Absorption coefficient	3.257 mm^{-1}	
F(000)	1360	
Crystal size	0.18 x 0.09 x 0.06 mm	
Data collection	Siemens SMART PLATFORM with CCD Detector<br>Graphite monochromator	
Detector distance	50 mm	
Method; exposure time/frame	omega-scans; t = 10 sec	
Solution by	direct methods	
Refinement method	full matrix least-squares on F^2, SHELXTL	
Theta range for data collection	2.46 to 30.05°	
Limiting indices	-15<=h<=13, -10<=k<=7, -32<=l<=34	
Reflections collected / unique	14217 / 2928 [R(int) = 0.0671]	
Completeness to θ = 29.13	99.9%	
Absorption correction	Semi-empirical from equivalents	
Max. and min. transmission	0.8412 and 0.5917	
Refinement method	Full-matrix least-squares on F^2	
Data / restraints / parameters	2928 / 0 / 154	
Goodness-of-fit on F^2	1.016	
Final R indices [I>2σ(I)]	R_1 = 0.0483, wR_2 = 0.0804	
R indices (all data)	R_1 = 0.0958, wR_2 = 0.0925	
Largest diff. peak and hole	1.823 and -1.078 e.$Å^{-3}$	

Selected intermolecular short contacts for 6

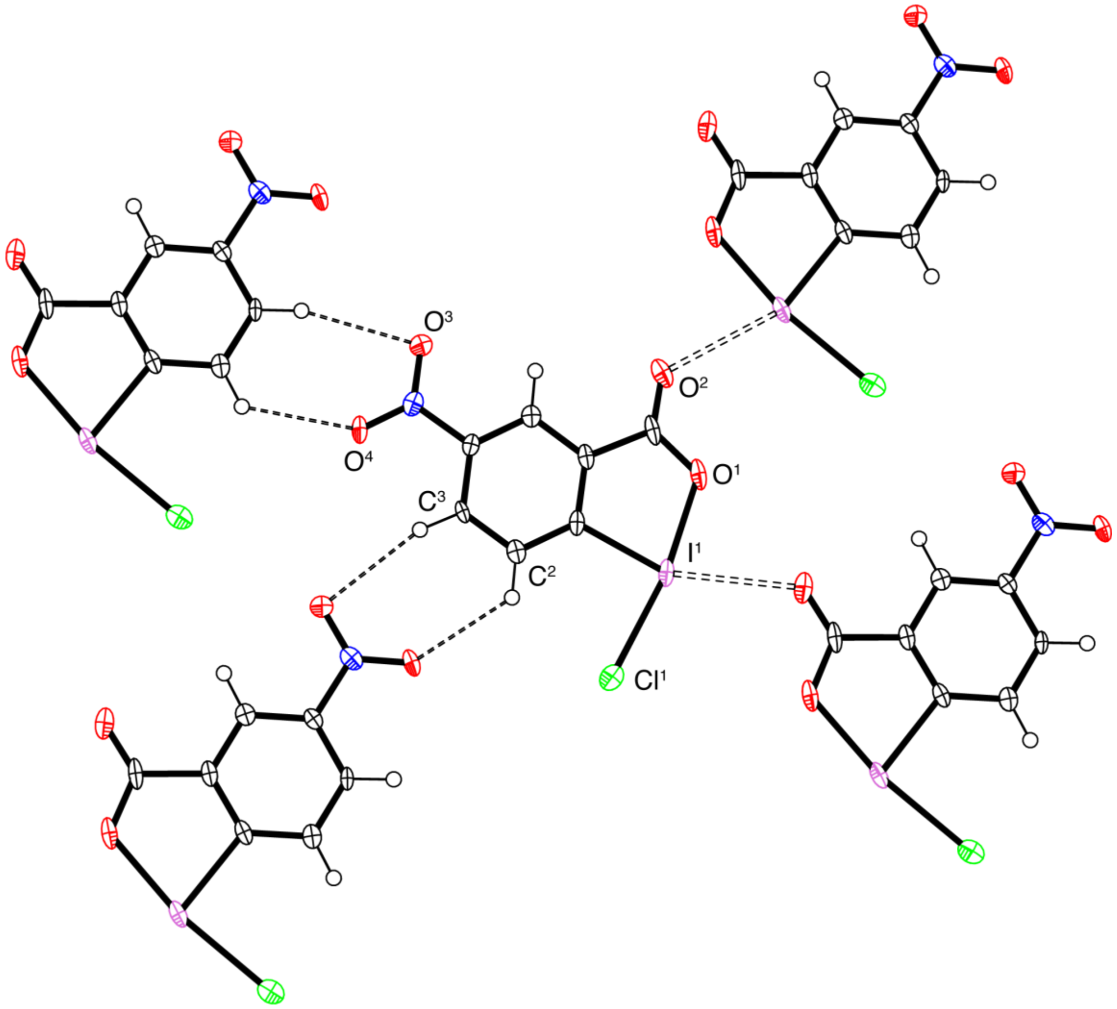

Figure S1: ORTEP representation with thermal ellipsoids set to 50% probability. Selected bond lengths (Å): C^2–O^4 2.46, C^3–O^3 2.56, I^1–O^2 2.84.

Selected intermolecular short contacts for 3

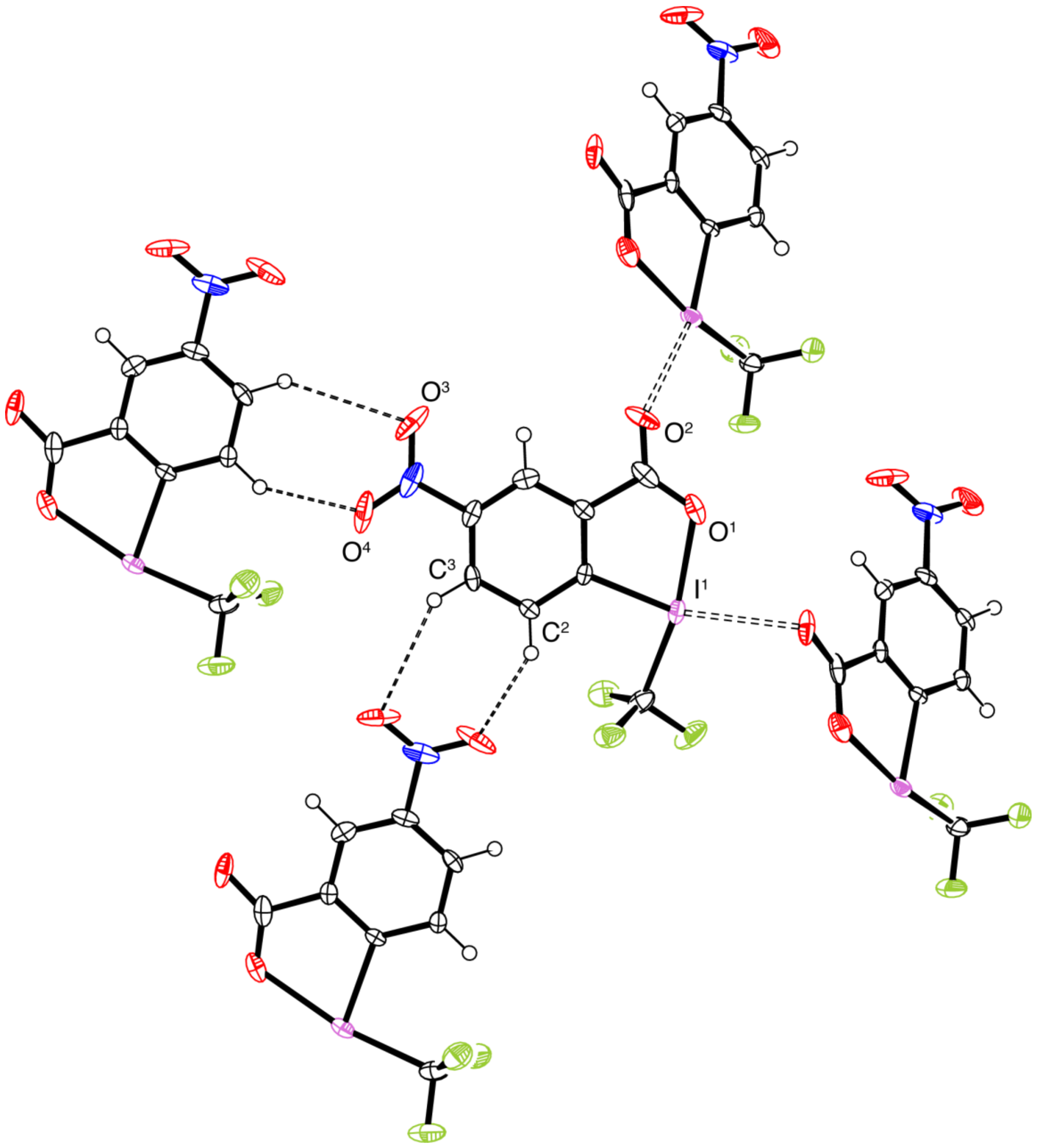

Figure S2: ORTEP representation with thermal ellipsoids set to 50% probability. Selected bond lengths (Å): C^2–O^4 2.41, C^3–O^3 2.92, I^1–O^2 2.76.

^{19}F NMR monitoring setup

All reactions were monitored by ^{19}F NMR spectroscopy using TopSpin on a Bruker Avance III HD Nanobay-400 spectrometer operating at 376 MHz. The experimental temperature was set to 298 K and maintained by a Bruker BCU II -80/60 temperature control unit. Initially, the temperature was equilibrated with a standard sample containing the appropriate reagent (**2**, **3**) and α,α,α-trifluorotoluene as internal standard. The shim was optimized using "topshim". The actual samples were prepared by mixing a reagent stock solution (**3**, 250 µL, 10.8 mM in MeCN-d_3 containing ~ 41 mM α,α,α-trifluorotoluene or **2**, 250 µL, 11.4 mM in MeCN-d_3 containing ~ 41 mM α,α,α-trifluorotoluene) and a stock solution of *p*-toluenesulfonic acid monohydrate (250 µL, 11.8 mM in MeCN) in a 0.5 mm NMR tube. The sample was then inserted into the magnet and the acquisition was started. Sequential ^{19}F NMR spectra measured every 60 s ("multi_zgvd" command, ds = 0, ns = 8) were used to monitor the reaction. Thus, a data point was obtained every 75 s along the reaction coordinate. The data obtained were processed (ef, phc0, phc1, pk, abs) using the batch processing command "multicmd" with phasing parameters determined for the last spectrum measured. Then, for every measuring point the integral for the signals of reagent and internal standard (α,α,α-trifluorotoluene) were determined and exported ("multi_integ3"). The exported data were then imported into R 2.14.0, a time scale was generated by multiplying the data row number by 75 s. In addition, for both reagents a blank spectrum only containing reagent and standard was measured ($I_{blank,\mathbf{2}}$ and $I_{blank,\mathbf{3}}$) in order to normalize the data. Intensity data were converted to concentrations according to $c(A) = 0.0055\ M \times (I(A)/I(S)) \times I_{blank}$, where c(A) refers to the concentration values of **2** or **3**, I(A) are the intensity values of **2** or **3** and I(S) the intensity values of the internal standard. I_{blank} refers to either $I_{blank,\mathbf{2}}$ or $I_{blank,\mathbf{3}}$. Every experiment was carried out at least 6 times to ensure reproducibility and allow for error estimation. As reagent **3** decayed exponentially, the rate constant $k_\mathbf{3}$ was obtained by considering $\ln([\mathbf{3}]) = -k_\mathbf{3} \times t + \ln([\mathbf{3}]_0)$. For reagent **2**, the approach of initial rates was applied and v_0 transformed to $k_\mathbf{2}$ under the assumption of an exponential decay ($k_2 = v_0/[\mathbf{2}]_0$). A summary of the data obtained is given in Table S1. Sample decays of **2** and **3** are depicted in Figure 3.

Table S1. Summary of rate data for the reaction of *p*-TsOH with **2**, **3**.

**Reagent 2**	**k_1 [s^{-1}]**	**k_2 [s^{-1}]**	**k_3 [s^{-1}]**	**k_4 [s^{-1}]**
	5.402 x 10^{-4}	6.120 x 10^{-4}	8.981 x 10^{-4}	3.931 x 10^{-4}
	**k_5 [s^{-1}]**	**k_6 [s^{-1}]**		
	5.919 x 10^{-4}	2.389 x 10^{-4}		

**Reagent 3**	**k_1 [s^{-1}]**	**k_2 [s^{-1}]**	**k_3 [s^{-1}]**	**k_4 [s^{-1}]**
	2.256 x 10^{-3}	4.427 x 10^{-3}	6.280 x 10^{-3}	5.984 x 10^{-3}
	**k_5 [s^{-1}]**	**k_6 [s^{-1}]**	**k_7 [s^{-1}]**	
	7.487 x 10^{-3}	2.191 x 10^{-3}	2.772 x 10^{-3}	

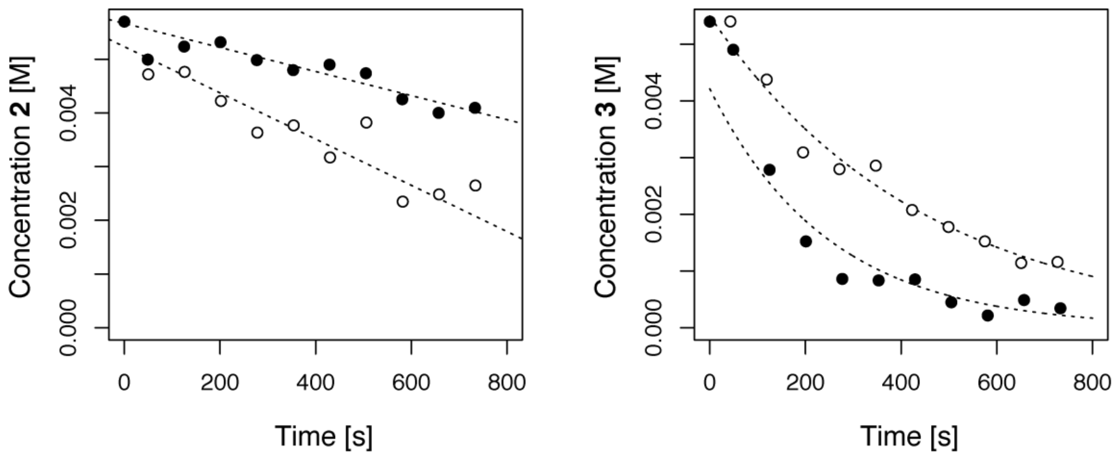

Figure S3: Sample decays of **2** (left) and **3** (right) and resulting fits (dashed lines).

Cyclic voltammetry spectrum of 3

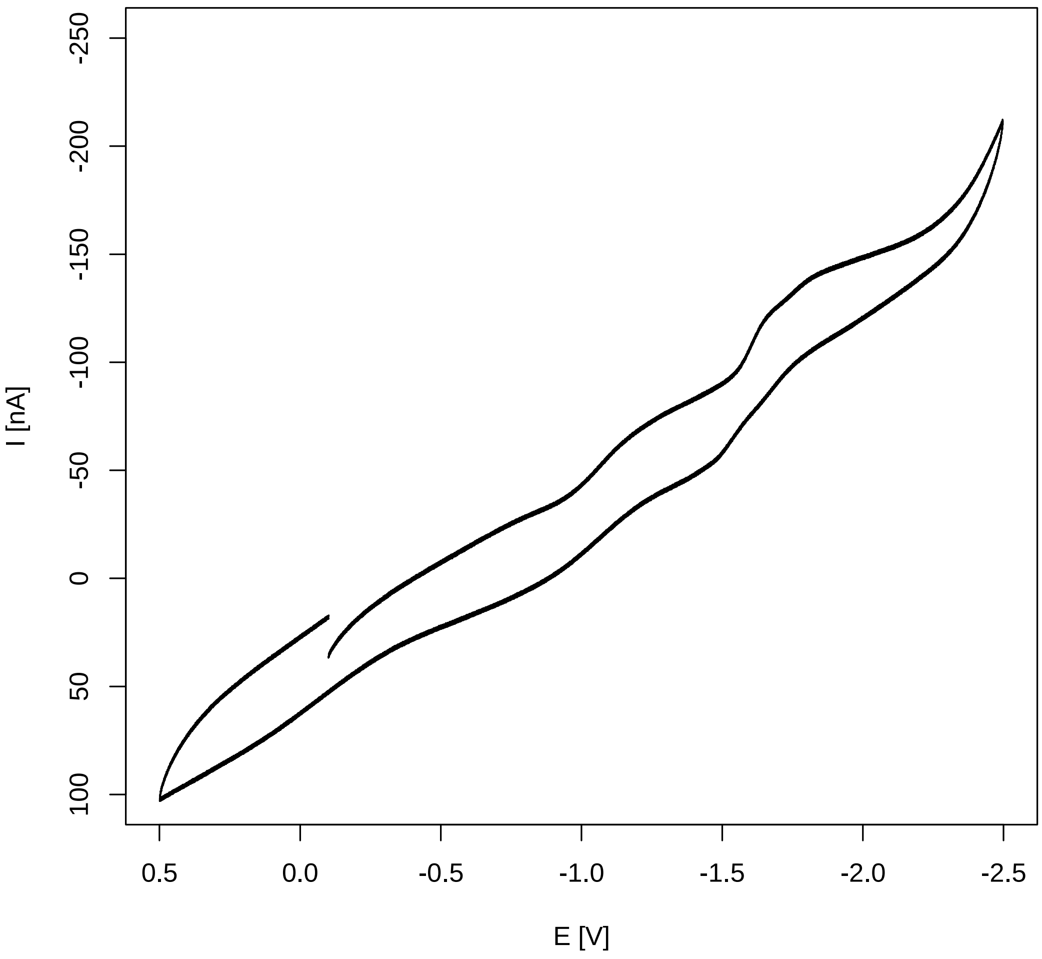

Figure S4: Unprocessed CV spectrum of reagent **3** (1 mM) in anhydrous MeCN + 0.1 M Bu_4NBF_4, platinum electrode, Ag/Ag^+ reference electrode, scan rate = 0.1 V/s.

References

1. Sheldrick, G. M. *Acta Crystallogr. A* **1990**, *46*, 467.
2. Sheldrick, G. M. Göttingen, Germany, **1999**.
3. Blessing, R. *Acta Crystallogr. A* **1995**, *51*, 33.
4. *Acta Crystallogr.* **2010**, *C66*, e1.
